# Intralesional nerve-sparing surgery versus non-surgical treatment for giant cell tumor of the sacrum

**DOI:** 10.1186/s12891-021-04907-0

**Published:** 2021-12-06

**Authors:** Shinji Tsukamoto, Nikolin Ali, Andreas F. Mavrogenis, Kanya Honoki, Yasuhito Tanaka, Paolo Spinnato, Davide Maria Donati, Costantino Errani

**Affiliations:** 1 Department of Orthopaedic Surgery, Nara Medical University, 840, Shijo-cho, Kashihara-city, Nara, 634-8521 Japan; 2 Department of Orthopaedic Oncology, IRCCS Istituto Ortopedico Rizzoli, Via Pupilli 1, 40136 Bologna, Italy; 3 First Department of Orthopaedics, School of Medicine, National and Kapodistrian University of Athens, 41 Ventouri Street, 15562 Holargos, Athens, Greece; 4 Radiology Unit, IRCCS Istituto Ortopedico Rizzoli, Via Pupilli 1, 40136 Bologna, Italy

**Keywords:** Giant cell tumor of bone, Sacrum, Denosumab, Embolization, Surgery, Intralesional nerve sparing surgery, Curettage

## Abstract

**Background:**

There is no standard treatment for giant cell tumors of the sacrum. We compared the outcomes and complications in patients with sacral giant cell tumors who underwent intralesional nerve-sparing surgery with or without (neo-) adjuvant therapies versus those who underwent non-surgical treatment (denosumab therapy and/or embolization).

**Methods:**

We retrospectively investigated 15 cases of sacral giant cell tumors treated at two institutions between 2005 and 2020. Nine patients underwent intralesional nerve-sparing surgery with or without (neo-) adjuvant therapies, and six patients received non-surgical treatment. The mean follow-up period was 85 months for the surgical group (range, 25–154 months) and 59 months (range, 17–94 months) for the non-surgical group.

**Results:**

The local recurrence rate was 44% in the surgical group, and the tumor progression rate was 0% in the non-surgical group. There were two surgery-related complications (infection and bladder laceration) and three denosumab-related complications (apical granuloma of the tooth, stress fracture of the sacroiliac joint, and osteonecrosis of the jaw). In the surgical group, the mean modified Biagini score (bowel, bladder, and motor function) was 0.9; in the non-surgical group, it was 0.5. None of the 11 female patients became pregnant or delivered a baby after developing a sacral giant cell tumor.

**Conclusions:**

The cure rate of intralesional nerve-sparing surgery is over 50%. Non-surgical treatment has a similar risk of complications to intralesional nerve-sparing surgery and has better functional outcomes than intralesional nerve-sparing surgery, but patients must remain on therapy over time. Based on our results, the decision on the choice of treatment for sacral giant cell tumors could be discussed between the surgeon and the patient based on the tumor size and location.

## Background

Giant bone tumor of bone (GCTB) is a locally aggressive, benign bone tumor with a high risk of local recurrence [1]. GCTB of the sacrum is very uncommon [2] and accounts for approximately 2% of all cases of GCTB [2]. Sacral GCTBs are often asymptomatic and cause symptoms only when they are considerably enlarged [3]. Sacral GCTBs usually occur in eccentric positions but can extend to both sides of the median line and anterior sacral space [4, 5]. It is close to important organs such as the large blood vessels, spinal cord, colon, and ureter; thus, surgery is difficult due to the complicated anatomy, and there is a high risk of massive bleeding during surgery. Most sacral GCTBs occur at the S1–2 levels [6], and wide resection, including the nerve roots of S1-S3, can reduce the local recurrence rate. However, it can cause severe functional losses, such as motor deficits and bowel, bladder, or sexual dysfunction, as well as lumbopelvic discontinuity [7]. Therefore, wide resection is usually unacceptable for the treatment of benign bone tumors [7]. Nerve-sparing surgery (also called intralesional curettage or piecemeal resection) can preserve the S1–3 nerve roots and maintain the stability of the pelvic ring, avoiding neurological deficits and lumbopelvic instability [8–10]. Although the recurrence rate is high, intralesional nerve-sparing surgery is recommended as a general surgical procedure for GCTBs [8–10]. Apart of local recurrence, intralesional nerve-sparing surgery could be associated with complications such as postoperative infection and massive bleeding during surgery [8–10].

The use of denosumab for GCTB was approved by the US Food and Drug Administration in 2013, and denosumab is indicated for GCTB that is inoperable or might cause severe dysfunction after surgery. It has been reported that the rate of disease control with denosumab therapy for inoperable GCTB is up to 96% [11]. However, complications such as osteonecrosis of the jaw, peripheral neuropathy, skin rash, hypophosphatemia, and atypical femoral fracture associated with long-term administration of denosumab have been reported [12]. Preoperative administration of denosumab makes curettage difficult and increases the risk of local recurrence [13]. Embolization has been performed for a long time for sacral GCTB, and a systematic review reported that the disease control rate is up to 75% [14]. Recently, Puri et al. reported that non-surgical treatment, which is a combination of denosumab therapy and embolization, was able to control disease progression in 11 of 12 patients (92%) with sacral GCTB during an average follow-up period of 31 months [15], and it has been proposed as a new treatment option for these tumors [15]. However, no study has compared the oncological and functional outcomes and complications between intralesional nerve-sparing surgery and non-surgical treatment (denosumab therapy and embolization) for sacral GCTB. We conducted this retrospective, comparative study in patients with GCTB of the sacrum to compare the oncological and functional outcomes and complications following intralesional nerve-sparing surgery and non-surgical treatment.

## Methods

We retrospectively investigated 16 cases of sacral GCTB treated at two institutions (IRCCS Istituto Ortopedico Rizzoli and Nara Medical University) between January 2005 and April 2020. One patient was excluded due to missing data, and the data of the remaining 15 patients were analyzed. Nine patients underwent intralesional nerve-sparing surgery with or without (neo-) adjuvant therapies (zoledronic acid, denosumab, or embolization), and six patients underwent non-surgical treatment (three patients received denosumab and embolization, and three patients received denosumab alone). We retrieved the following data from the patients’ medical records: age; sex; tumor size measured by computed tomography (CT) or magnetic resonance imaging (MRI); anatomical level of the tumor; Campanacci stage [2]; tumor involvement of the sacroiliac joint; involvement of the vascular or other organ systems; location; spinal instability (spinopelvic stability was considered intact if at least the cephalad 50% of the S-1 vertebra and sacroiliac joints were preserved bilaterally [16]); surgical approach; reconstruction; local recurrence or tumor progression; treatment for local recurrence; neurological status and pain before and after treatment; lung metastasis; oncological outcome; complications related to surgery, denosumab, zoledronic acid, or embolization; Karnofsky performance status; and evaluation of bowel, bladder, and motor function using modified Biagini score (Table 1) [17]. For female patients, we also collected data on whether they were pregnant or delivered a baby after developing sacral GCTB and their follow-up period. The follow-up period (mean, 59 months; range, 17–94 months) of the non-surgical treatment group was shorter than that of the intralesional nerve-sparing surgery group (mean, 85 months; range, 25–154 months) (Table 2). There was no difference between the two groups in terms of clinical symptoms and staging at presentation: all 15 patients had pain and Campanacci stage III tumor at presentation. In the intralesional nerve-sparing surgery group, the mean tumor volume was 111 cm^3^ (range 14–235), the tumor level was above S3 in 33% of the patients, at or below S3 in 11% of the patients, and involved the whole sacrum in 56% of the patients. Tumor involvement of the sacroiliac joint was observed in 56% of patients, tumor involvement of the vascular or other organ systems was observed in 56% of patients, and the tumor was located centrally in 22% of patients (Table 2). In the non-surgical treatment group, the mean tumor volume was 272 cm^3^ (range 99–678), the tumor level was above S3 in 17% of patients, and it involved the whole sacrum in 83% of patients. Tumor involvement of the sacroiliac joint was not observed, tumor involvement of the vascular or other organ systems was observed in 17% of patients, and the tumor was located centrally in 83% of patients (Table 2).

**Table 1:** Modified Biagini score (classification of neurologic function after resection of the sacrum) [17]

Function	Score	Description
Bladder	0	Normal
	1	Feels stimulus to micturate and has limited continence at varying times and quantities of urine and/or has increasing postmicturition vesical residual and/or urinary loss in conditions of stress
	2	Does not feel stimulus to micturate and/or is completely incontinent
Bowel	0	Normal
	1	Feels stimulus to defecate and is incontinent when feces are soft or under stress
	2	Does not feel stimulus to defecate and/or is completely incontinent
Motor	0	Normal or mild deficit not requiring the help of external support for motion and common activities
	1	Deficits requiring the help of external support for walking and common activities
	2	Deficits that make walking impossible

**Table 2:** Patients’ characteristics and outcomes in the nerve-sparing surgery and non-surgical treatment groups

	Nerve-sparing surgery group (n = 9)	Non-surgical treatment group (*n* = 6)
Age (years)	Mean 29 (range, 15–48)	Mean 40 (range, 14–66)
Sex		
Male	2 (22%)	2 (33%)
Female	7 (78%)	4 (67%)
Tumor volume (cm^3^)	Mean 111 (range, 14–235)	Mean 272 (range, 99–678)
Tumor level		
Above S3	3 (33%)	1 (17%)
At or below S3	1 (11%)	0
At both levels	5 (56%)	5 (83%)
Involvement of the sacro-iliac joint		
No	4 (44%)	6 (100%)
Yes	5 (56%)	0
Involvement of the vascular or other organ system		
No	4 (44%)	5 (83%)
Yes	5 (56%)	1 (17%)
Location		
Central	2 (22%)	5 (83%)
Eccentric	7 (78%)	1 (17%)
Local recurrence or tumor progression		
No	5 (56%)	6 (100%)
Yes	4 (44%)	0
Lung metastasis		
No	8 (89%)	6 (100%)
Yes	1 (11%)	0
Oncological outcome		
CDF	5 (56%)	0
NED	1 (11%)	0
AWD	3 (33%)	6 (100%)
Complications		
None	5 (56%)	5 (83%)
Infection	1 (11%)	0
Bladder laceration	1 (11%)	0
Stress fracture of the sacro-iliac joint	1 (11%)	0
Apical granuloma of the tooth	1 (11%)	0
Osteonecrosis of the jaw	0	1 (17%)
Karnofsky performance status	Mean 87 (range, 65–95)	Mean 88 (range, 75–100)
Total of modified Biagini score	Mean 0.9 (range, 0–4)	Mean 0.5 (range, 0–2)
Follow-up (months)	Mean 85 (range, 25–154)	Mean 59 (range, 17–94)

CDF, continuous disease free; NED, no evidence of disease; AWD, alive with disease

Intralesional nerve-sparing surgery was indicated in patients who had tumors located eccentrically. In the intralesional nerve-sparing surgery group, preoperative denosumab therapy (weekly for the first month, then once a month for a total of 10 cycles) was administered in 3 cases, preoperative embolization was performed in 1 case, preoperative zoledronic acid (once a month for a total of 2–6 cycles) and embolization was performed in 3 cases, and the remaining 2 patients did not receive any preoperative adjuvant treatment (Table 3). Surgery after the end of administration of denosumab and zoledronic acid was scheduled before the start of drug administration. Preoperative embolization was performed within 48 h prior to surgery. Seven cases were operated using the posterior approach, and two cases were operated using the anterior/posterior approach (Table 3). The indications for an anterior approach were large tumors with anterior extraosseous lesions. Through the anterior approach, we ligated the hypogastric, internal iliac, and tumor vessels and separated the tumor from the rectum. Through the posterior approach, we performed a wide laminectomy and complete curettage with a curette and high-speed burr. Sacral nerve roots were identified and preserved. The bilateral nerve roots of S1–3 were preserved using curettage. Phenol was used as a local adjuvant therapy in six patients but not in areas close to the sacral nerve roots (Table 3) [6].

**Table 3:** Details of the 15 patients with sacral giant cell tumors

Case	Age (years)	Sex	Tumor volume (cm^3^)	Tumor level	Involvement of the sacro-iliac joint	Involvement of the vascular or other organ system	Location	Preoperative treatment	Surgical approach	Local adjuvant therapy	Local recurrence or tumor progression	Treatment for local recurrence
Nerve-sparing surgery group												
1	35	F	91	S2-S4	Yes	None	Eccentric	Denosumab (pre-op: 10 cycles and post-op: every 3 months)	Posterior	None	Yes (3 years after surgery)	Embolization and curettage
2	36	F	14	S2-S3	No	None	Eccentric	None	Posterior	Phenol	Yes (52 months after surgery)	Denosumab (monthly for 10 months)
3	48	F	75	S1-S2	Yes	None	Eccentric	Embolization and zoledronate (2 cycles)	Posterior	Phenol	Yes (53 months after surgery)	Denosumab (monthly for a year) and curettage
4	16	F	14	S1-S3	Yes	Involvement of sacral plexus	Eccentric	Embolization	Posterior	Phenol	No	NA
5	30	F	80	S1-S2	No	None	Eccentric	Denosumab (10 cycles)	Posterior	Phenol	No	NA
6	43	M	295	S3-S5	No	Rectum compression	Central	None	Posterior	None	No	NA
7	15	M	62	S1-S2	Yes	None	Eccentric	Embolization (three times) and zoledronate (one cycle)	Posterior	Phenol	No	NA
8	15	F	132	S1-S4	No	Rectum and uterus compression	Central	Denosumab (pre-op: 10 cycles and post-op: 6 cycles)	Anterior/ posterior	None	Yes (14 months after surgery)	Denosumab (every 6 months for 5 years and 10 months)
9	19	F	235	S1-S3	Yes	Rectum compression	Eccentric	Embolization and zoledronate (pre-op: 6 cycles and post-op: 6 cycles)	Anterior/ posterior	Phenol	No	NA
Non-surgical treatment group												Duration and interval of the denosumab administration
10	14	F	99	S1-S3	No	Uterus compression	Central	NA	NA	NA	No	Monthly for 5 years and bimonthly for 2 years
11	32	M	230	S2-S5	No	None	Central	NA	NA	NA	No	Monthly for 3 years and every 3 months for 4 months
12	66	F	678	S1-S4	No	None	Central	NA	NA	NA	No	Monthly for 3 years and every 3 months for 4 years
13	65	F	146	S1-S4	No	None	Central	NA	NA	NA	No	Monthly for 3 years and every 3 months for 27 months
14	31	M	252	S1-S2	No	None	Central	NA	NA	NA	No	Monthly for a year and then bimonthly for 4 months
15	29	F	226	S1-S4	No	None	Eccentric	NA	NA	NA	No	Monthly for a year

NA, not applicable; M, male; F, female

**Fig. 1:**
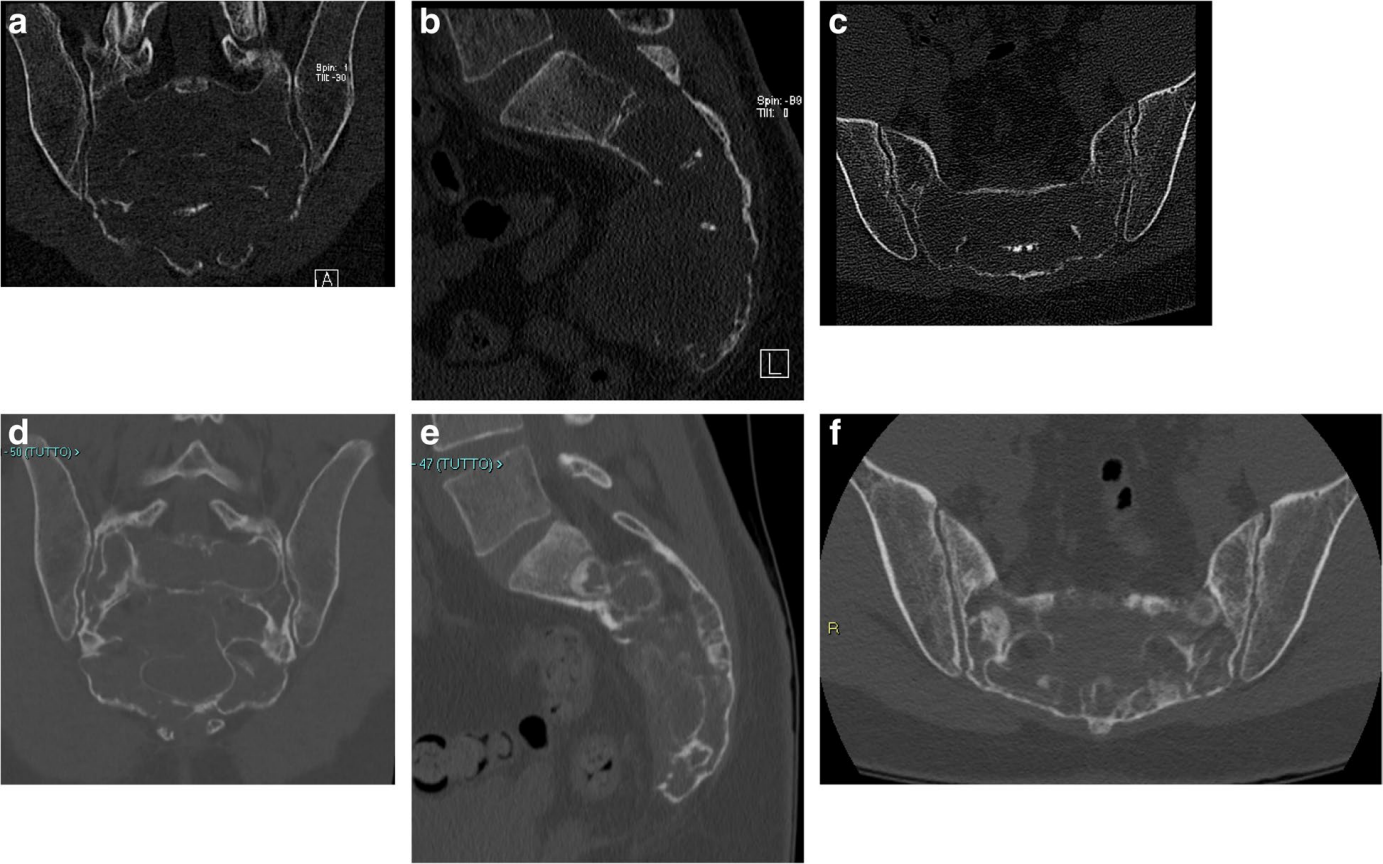
A case of sacral giant cell tumor of bone treated with no surgical treatment (denosumab alone) (Case 11). Computed tomography at presentation showed an osteolytic lesion of the sacrum (a: coronal view, b: sagittal view, c: axial view). Computed tomography showed bone sclerosis 3 years after the diagnosis, after 40 doses of denosumab treatment (d: coronal view, e: sagittal view, f: axial view)

Non-surgical treatment (denosumab therapy or embolization) was indicated for patients in whom large tumors were centrally located. Denosumab 120 mg was administered subcutaneously to all six patients once a month for 1–5 years (weekly for the first month) and then every 2–3 months (Table 3). The patients also received daily calcium (2500 mg) and vitamin D (≥ 400 IU). Surgery was not scheduled before the start of denosumab administration. Embolization was performed in 3 of the 6 patients. It was performed once a month for a total of three times in one of the three patients (case 10) and, in the remaining two cases, every three months for a total of two and three times (Cases 14 and 15, respectively) (Table 3). Embolization was discontinued when the hypervascular tumor disappeared, no tumor growth was observed on imaging, and the clinical symptoms improved. Intra-arterial embolization was performed using femoral access to selectively embolize the main arteries feeding the tumor. Angiography was performed at the beginning of each treatment session to identify arteries of adequate caliber to facilitate embolization. The arteries were embolized based on the arterial supply to the sacrum, resulting in occlusion of the internal iliac, lateral sacral, and median sacral arteries. Selective delivery of substances, including embosphere microspheres or gelatin sponges, was used to achieve central occlusion of the vessels. Postprocedural angiography showed complete interruption of the tumor blood supply and more than 80% devascularization of the tumor in all cases (Fig. 1).

Routine follow-up evaluation was performed every 3 months for the first 3 years, every 6 months for the next 2 years, and then annually. Each follow-up evaluation included assessment of sexual dysfunction, clinical examination of motor, sensory, bladder, and bowel deficits, and imaging evaluation, including CT or MRI of the pelvis. Chest CT was performed annually [6]. Postoperative local recurrence was defined as bone resorption, expansile osseous destruction, or local soft tissue mass formation on CT and MRI. Tumor progression during non-surgical treatment was defined as a new area of ​​osteolysis or new cortical destruction on CT and MRI [18].

The independent ethics committee of each institution approved the study. Informed consent was obtained from all individual participants in IRCCS Istituto Ortopedico Rizzoli, and the requirement for written consent from participants in Nara Medical University was waived, because an “opt-out” process was used and the study had the retrospective nature.

## Results

The local recurrence rate was 44% (4 of 9 patients) in the intralesional nerve-sparing surgery group, whereas the tumor progression rate was 0% (none of 6 patients) in the non-surgical treatment group. The lung metastasis rate was 11% (1 of 9 patients) in the intralesional nerve-sparing surgery group and 0% (none of 6 patients) in the non-surgical treatment group. The patient with lung metastasis received neo- and adjuvant denosumab therapy. Six of nine patients (67%) in the intralesional nerve-sparing surgery group achieved a disease-free status. In the intralesional nerve-sparing surgery group, complications occurred in 44% of the patients (4 of 9 patients): 1 case each of postoperative infection, intraoperative bladder laceration, stress fracture of the sacroiliac joint, and denosumab-related apical granuloma of the tooth, whereas denosumab-related osteonecrosis of the jaw occurred in 1 patient (17%) (1 of 6 patients) in the non-surgical treatment group. There were no complications related to zoledronic acid or embolization. In the intralesional nerve-sparing surgery group, the Karnofsky performance status was 87 (range, 65–95), whereas in the non-surgical treatment group, it was 88 (range, 75–100). In the intralesional nerve-sparing surgery group, the mean modified Biagini score was 0.9 (range, 0–4), whereas in the non-surgical treatment group, it was 0.5 (range, 0–2) (Table 2).

None of the 15 patients had spinal instability or required reconstruction. None of the patients underwent radiotherapy or malignant transformation. Of the four patients who experienced local recurrence following intralesional nerve-sparing surgery, one underwent embolization and re-curettage, one underwent denosumab therapy and re-curettage, and the remaining two received denosumab therapy with which the disease remained stable (Table 3). In the intralesional nerve-sparing surgery group, two of the three patients (67%) who received preoperative or pre- and postoperative denosumab therapy experienced local recurrence, whereas two of six patients (33%) who did not receive preoperative denosumab therapy experienced local recurrence (Table 3). The details of the 15 cases are presented in Tables 3 and 4. None of the 11 female patients became pregnant or delivered a baby after the development of sacral GCTB.

**Table 4:** Details of the 15 patients with sacral giant cell tumors

Case	Neurological status before treatment	Neurological status after treatment	Lung metastasis	Oncological outcome	Complications	Karnofsky performance status	Modified Biagini score	Follow-up (months)
Nerve-sparing surgery group								
1	Hypotonia of the lower limb, urinary and fecal incontinence, and local pain	Paralysis in the S1-S3 regions, urinary retention, and severe local pain	Yes	AWD	Infection. Treatment: Debridement three times and antibiotic agents	65	Bl-2, Bo-1, Mo-1	96
2	Left-sided sciatica	Paresthesia in the right S2 region	No	AWD	None	90	Bl-0, Bo-0, Mo-0	63
3	Constipation and local pain	Perianal hypoesthesia, residual voiding, constipation, and slight local pain (improved over time)	No	NED	Apical granuloma of the tooth occurred 14 months after discontinuing denosumab.	90	Bl-1, Bo-0, Mo-0	154
4	Sciatica and local pain	Slight pain	No	CDF	None	95	Bl-0, Bo-0, Mo-0	63
5	Right-sided sciatica and local pain	Local pain	No	CDF	None	90	Bl-0, Bo-0, Mo-0	25
6	Local pain	Dysuria	No	CDF	None	95	Bl-0, Bo-0, Mo-0	84
7	Left-sided sciatica	Slight pain	No	CDF	None	95	Bl-0, Bo-0, Mo-0	46
8	Local pain and urinary retention	Occasional urinary incontinence	No	AWD	Stress fracture of the sacro-iliac joint: Treatment: Oral painkillers	80	Bl-1, Bo-0, Mo-0	100
9	Local pain and urinary incontinence	Anal sphincter deficiency (manual evacuation), weakness of the right triceps surae muscle	No	CDF	Bladder laceration. Treatment: Immediately repaired	80	Bl-0, Bo-2, Mo-0	132
Non-surgical treatment group								
10	Bilateral sciatica and urinary incontinence	Mild pain in the S1 region	No	AWD	None	75	Bl-1, Bo-1, Mo-0	89
11	Paresthesia in the S1-S2 region, dysuria and dyschezia	Symptoms are relieved	No	AWD	None	100	Bl-0, Bo-0, Mo-0	54
12	Local pain	No pain	No	AWD^a^	Osteonecrosis of the jaw occurred 6 years after starting denosumab.^b^	80	Bl-1, Bo-0, Mo-0	94
13	Local pain	Slight pain	No	AWD	None	90	Bl-0, Bo-0, Mo-0	77
14	Local pain, left-sided sciatica, and loss of dorsiflexion of the right toe	Slight pain	No	AWD	None	90	Bl-0, Bo-0, Mo-0	22
15	Local pain, right-sided sciatica, urinary retention, and muscle weakness of the lower limbs	No pain, no sphincter disorders, and no muscle weakness	No	AWD	None	90	Bl-0, Bo-0, Mo-0	17

CDF, continuous disease free; NED, no evidence of disease; AWD, alive with disease; Bl, bladder; Bo, bowel; Mo, motor

^a^Now the disease remains stable without denosumab therapy. ^b^Denosumab was discontinued, jaw surgery was performed, and denosumab was resumed two months later

## Discussion

The recurrence rate in the intralesional nerve-sparing surgery group was higher than that in the non-surgical treatment group; however, 67% of the patients (6 of 9 patients) in the intralesional nerve-sparing surgery group achieved disease-free status. According to the literature, the local recurrence rate of intralesional nerve-sparing surgery was 0–100% [6–10, 16, 19–31], and the local recurrence rate of intralesional nerve-sparing surgery combined with preoperative denosumab therapy was 11–67% [9, 10, 22, 29, 30, 32] (Table 5). The combination of denosumab therapy and embolization led to stable disease in 42–100% of patients [3, 15]. Embolization alone showed a response in 67–82% of the patients and led to stable disease in 50% of patients [33–37]. Bisphosphonate alone showed a response in 11% of the patients, leading to stable disease in 67% of the patients and disease progression in 22% of the patients [38] (Table 6). Our results confirm the previous data in the literature that the local recurrence rate after intralesional nerve-sparing surgery appears to be higher than the disease progression rate after non-surgical treatment.

**Table 5:** Overview of studies reporting the result of nerve sparing surgery in sacral giant cell tumor

Nerve sparing surgery without preoperative denosumab treatment									
First author, year of publication	Tumor level	Campanacci stage	Local adjuvant therapy	Interval between the first surgery and local recurrence (months)	Number of patients	Local recurrence	Follow-up (months)	Functional outcome	Complications
Balke, 2012 [20]	NR	NR	Post-op RT: 30%	Mean 12	10	2 (20%)	Mean 52	NR	Infection: 10%
Chen, 2015 [21]	Above S3: 25%; at or below S3: 25%; in both parts: 50%	NR	Zoledronic acid-loaded cement: 100%	NA	4	0	Mean 28	Improved: 100%	None
Chen, 2018 [22]	NR	NR	NR	NR	10	3 (30%)	NR	NR	NR
Domovitov, 2016 [16]	Above S3: 0%; at or below S3: 8%; in both parts: 92%	Stage 1: 8%; stage 2: 21%; stage 3: 71%	Pre-op RT: 54%; post-op RT: 4%; liquid nitrogen: 79%	NR	24	7 (30%)	Mean 86	Improved: 79%; stable: 13%; worsen: 8%	Infection: 21%; Skin necrosis: 13%; Rectal fistula: 4%; Avascular necrosis: 8%; Stress fracture due to RT: 8%; Malignant transformation: 4%
Guo, 2009 [8]	Above S3: 4; at or below S3: 2; in both parts: 18	Stage 2: 79%; stage 3: 21%	Post-op RT: 8%	Mean 13	24	7 (29%)	Mean 58	All the patients were able to walk without an assistive device. Seventeen (70.8%) patients retained normal urinary function and 16 (66.7%) patients preserved normal bowel function.	Infection: 25%; Cerebrospinal fluid leakage: 21%; Deep-vein thrombosis: 4%
Kollender, 2003 [23]	NR	NR	Cryosurgery: 100%	NA	3	0	Mean 61	NR	Infection: 33%
Li, 2012 [24]	Above S3: 38%; at or below S3: 6%; in both parts: 56%	NR	Post-op RT: 25%	NR	32	12 (38%)	Median 42	Five patients (15.6%) developed urinary bladder dysfunction and two patients (6.3%) developed bowel dysfunction requiring medication. Four patients with marginal resections had lower limb dysfunction (12.5%).	Malignant transformation: 6%, Infection: 34%
Lim, 2020 [9]	S1 involvement: 78%	Stage 3: 100%	NR	NR	36	12 (33%)	NR	Mean MUD score increased from 23.9 preoperatively to 25.4 postoperatively.	NR
Martin, 2010 [25]	Above S3: 50%; at or below S3: 0%; in both parts: 50%	NR	Post-op RT: 50%	Mean 7	6	2 (33%)	Mean 34	Normal: 80%, pain and fecal incontinence: 20%	NR
Ruggieri, 2010 [6]	Above S3: 32%; at or below S3: 6%; in both parts: 61%	Stage 2: 3%, stage 3: 97%	Pre-op RT: 3%; post-op RT: 65%; phenol: 45%; liquid nitrogen: 3%	Within 34	31	3 (10%)	Median 108	The incidence of L5-S2 neurologic deficits decreased from 23% preoperatively to 13% postoperatively. The incidence of S3-S4 neurological deficits increased from 16% preoperatively to 33% postoperatively.	Infection: 26%; Massive bleeding: 23%
Sung, 1982 [31]	S1–3: 100%	NR	NR	8	2	1 (50%)	84	NR	NR
Turcotte, 1993 [19]	Above S3: most frequent	NR	RT: 81%	NR	17	17 (100%)	Mean 94	Improved: 53%; stable: 35%; worsened: 12%	Malignant transformation: 18%; Death due to massive bleeding: 6%
Thangaraj, 2010 [7]	Above S3: 13%; at or below S3: 13%; in both parts: 75%	NR	NR	Mean 16	8	3 (38%)	Mean 152	Improved: 25%, stable: 38%, worsen: 38%	Massive bleeding: 25%; Infection: 13%; RT-induced menopause: 13%
van der Heijden, 2014 [26]	Above S3: 58%; at or below S3: 4%; in both parts: 38%	NR	RT: 19%; phenol: 15%; liquid nitrogen: 35%; argon beam coagulation: 12%	Median 13	26	14 (54%)	Median 98	Median MSTS 24	Massive bleeding: 15%; Infection: 12%; Drop foot: 12%; Hardware failure: 4%; RT-induced sarcoma: 4%; Fracture due to RT: 4%
Wang, 2020 [27]	Above S3: 27%; at or below S3: 9%; in both parts: 64%	Stage 2: 18%; stage 3: 82%	NR	NR	11	5 (45%)	Mean 60	Normal: 64%, urinary and fecal incontinence: 27%, bowel obstruction: 9%	Infection: 36%; Thrombosis due to the aortic balloon occlusion: 9%
Xu, 2017 [28]	Above S3: 19%; at or below S3: 0%; in both parts: 81%	Stage 2: 13%; stage 3: 88%	RT: 38%	Mean 15	16	7 (44%)	Mean 92	Normal: 56%	NR
Yang, 2018 [29]	Above S3: 100%	Stage 3: 100%	NR	NA	10	0	Mean 35	Mean MSTS: 73%	NR
Zhao, 2020 [10]	Above S3: 24%; at or below S3: 6%; in both parts: 70%	Stage 2: 18%; stage 3: 82%	NR	NR	89	26 (29%)	Median 58	NR	NR
Nerve sparing surgery combined with preoperative denosumab									
First author, year of publication	Level	Campanacci stage	Number of patients	Local recurrence	Preoperative denosumab	Postoperative denosumab	Follow-up (months)	Functional outcome	Complications
Chen, 2018 [22]	NR	NR	10	2 (20%)	1–11 doses	4–24 doses (9 patients)	NR	NR	Osteonecrosis of the jaw: 0%; Malignant transformation: 10%
Lim, 2020 [9]	S1 involvement: 94%	Stage 3: 100%	17	3 (18%)	1–4 doses	Mean 14.8 doses (16 patients)	NR	Mean MUD score increased from 23.9 preoperatively to 25.4 postoperatively.	Malignant transformation: 6%
Niu, 2019 [32]	NR	Stage 3: 100%	6	3 (50%)	3–12 months	None	Mean 19	NR	NR
Wang, 2020 [27]	Above S3: 25%; at or below S3: 0%; in both parts: 75%	Stage 2: 25%; stage 3: 75%	4	0	NR	NR	Mean 36	Normal: 75%; urinary incontinence and bowel obstruction: 25%	Infection: 25%
Xu, 2017 [28]	Above S3: 21%; at or below S3: 0%; in both parts: 79%	Stage 2: 32%; stage 3: 68%	19	2 (11%)	1dose bisphosphonate	2 years bisphosphonate 1 dose at 4-weeks intervals	Mean 47	Normal: 89%	NR
Yang, 2018 [29]	Above S3: 100%	Stage 3: 100%	6	4 (67%)	Mean 5.2 months	None	Mean 12	Mean MSTS 87%	NR
Zhang, 2019 [30]	S1–3: 67%; S2–4: 33%	Stage 3: 100%	3	2 (67%)	6 doses	None	Mean 38	NR	NR
Zhao, 2020 [10]	Above S3: 24%; at or below S3: 6%; in both parts: 70%	Stage 2: 18%; stage 3: 82%	19	6 (32%)	1–4 doses	2–30 doses (18 patients)	Median 58	NR	NR

NR, not reported; NA, not applicable; MUD, Motor function and sensation of lower limb (M) Urination and uresiesthesia (U) Defecation and rectal sensation (D); RT, radiotherapy; MSTS, musculoskeletal tumor society

**Table 6:** Studies reporting the result of non-surgical treatments excluding radiotherapy in sacral giant cell tumor

First author, year of publication	Tumor level	Campanacci stage	Number of patients	Response	Follow-up (months)	Functional outcome	Complications
Denosumab combined with embolization							
Ji, 2017 [3]	S1–4	Stage 3: 100%	1	Stable: 100%	31	Asymptomatic: 100%	None
Puri, 2020 [15]	S1: 77%; S2: 15%; S3: 8%	Stage 3: 100%	12	Stable: 42%; Progression: 58%	Mean 49	10 patients (83%) were asymptomatic. The patient with loss of bladder control at presentation recovered.	Foot drop: 17%
Embolization							
Chuang, 1981 [33]	NR	NR	3	Response: 67%	Mean 34	2 patients (67%) recovered from pain	Foot drop: 33%, Foot numbness: 33%
Hosalkar, 2007 [34]	Above S3: 0%; at or below S3: 0%; in both parts: 100%	stage 2: 67%; stage 3: 33%	9	Partial response: 78%; progression: 22%	Mean 108	Mean MSTS 29	NR
Lackman, 2002 [35]	NR	NR	4	Stable: 50%; progression: 50%	Mean 80	All the patients (100%) recovered from pain.	NR
Lin, 2002 [36]	Above S3: 50%, at or below S3: 33%, in both parts: 17%	NR	17	Partial response: 82%; progression: 18%	Median 105	14 patients (73%) recovered from pain and neurologic symptoms.	Foot drop: 12%, Foot numbness: 6%; Malignant transformation due to RT: 12%
Nakanishi, 2013 [37]	NR	NR	4	Partial response: 75%; progression: 25%	Mean 78	Mean MSTS increased from 28% preoperatively to 90% postoperatively.	Foot drop: 25%
Bisphosphonate							
Balke, 2010 [38]	NR	NR	9 (3 patients underwent surgery; 1 received interferon therapy, 2 received RT, 7 underwent embolization)	Partial response: 11%; stable: 67%; progression: 22%	Mean 24	NR	None

NR, not reported; RT, radiotherapy; MSTS, musculoskeletal tumor society

The effects of embolization include pain relief, reduced vascularity, and peripheral ossification on radiographs [7, 39]. Typical embolization intervals have been reported to be 4–6 weeks [39, 40]. Lin et al. reported that the local recurrence rate following embolization for sacral GCTB was 31% at 10 years and 43% at 20 years [36]. Lackman et al. reported 5 cases of sacral GCTB treated with embolization alone; the tumor size remained stable in four patients (80%) after an average of 6.7 years of follow-up [35]. According to a systematic review by He et al. [14], during a mean follow-up period of 86 months, the frequency of embolization ranged from 1 to 10 times (mean, 4.1 times). All 44 patients were responsive to embolization, and the objective radiographic response rate was 82% (36/44) [14]. The 2-, 5-, and 10-year local control rates were 93% (41/44), 91% (40/44), and 82% (36/44), respectively [14]. Puri et al. reported the outcomes of 13 patients with sacral GCTB who underwent non-surgical treatment consisting of denosumab, embolization, and radiotherapy [15]. Patients were evaluated every 10–12 weeks, and no further treatment was recommended once the tumor stopped growing [15]. If the tumor grew, denosumab was added and/or embolization was performed until local control of the tumor was achieved [15]. Tumor growth was stopped in 12 of the 13 patients (92%) [15]. The total number of embolizations ranged from 0 to 12 (mean = 4). The total number of denosumab doses ranged from 5 to 16 (mea*n* = 9) [15]. Eight of the 13 patients received radiotherapy [15]. One patient with bladder dysfunction at presentation recovered during the treatment [15]. Two patients experienced transient weakness in ankle dorsiflexion due to embolization, but this spontaneously relieved [15].

The results of our study showed that there were two surgery-related complications (infection and bladder laceration) and three denosumab-related complications (apical granuloma of the tooth and stress fracture of the sacroiliac joint in the intralesional nerve-sparing surgery group and osteonecrosis of the jaw in the non-surgical treatment group). According to the literature, complications associated with intralesional nerve-sparing surgery include infection in 10–36% of patients [6–8, 16, 20, 23, 24, 26, 27], skin necrosis in 13% [16], rectal fistula in 4% [16], avascular necrosis in 8% [16], cerebrospinal fluid leakage in 21% [8], deep vein thrombosis in 4% [8], massive bleeding in 6–23% [6, 7, 26], drop foot in 12% [26], hardware failure in 4% [26], and thrombosis in 9% [27] (Table 5). Complications associated with nerve-sparing surgery following preoperative denosumab therapy were infection in 25% [27] and malignant transformation in 6–10% of the patients [9, 22] (Table 5). Complications associated with embolization were foot drop in 12–33% of the patients [15, 33, 36, 37] and foot numbness in 6–33% [33, 36] (Table 6). According to a systematic review by He et al. [14], the incidence of neurological complications following embolization was 14% (6/44). None of the patients experienced bowel, bladder, or sexual dysfunction due to embolization [14]. No complications were associated with bisphosphonate use alone [38] (Table 6). Contrary to our results, the literatures showed that the frequency of complications associated with intralesional nerve-sparing surgery appears to be higher than that with non-surgical treatment.

Tang et al. reported that sacral tumors located in S1–2 or those larger than 200 cm^3^ in volume had a higher risk of massive bleeding during surgery [41]. Lim et al. reported that preoperative denosumab administration could reduce surgical time by reducing bleeding [9]. According to the results of a phase 2 study of denosumab for GCTB, during the treatment phase, the most common grade 3 or higher adverse events were hypophosphatemia (24 [5%] of 526 patients), osteonecrosis of the jaw (17 [3%], pain in extremities [12 [2%]), and anemia (11 [2%]) [42]. Four (1%) patients had atypical femur fractures, and four (1%) had hypercalcemia occurring 30 days after denosumab discontinuation [42]. There were 4 cases (1%) of malignant transformation, consistent with historical data [42].

In our study, although non-surgical treatment was more frequently performed for larger GCTBs that were centrally located in the sacrum, the Karnofsky performance status was similar in both groups (mean 87 vs. 88 in the intralesional nerve-sparing surgery and non-surgical treatment groups, respectively), and the total modified Biagini score was better in the non-surgical treatment group (mean 0.5) than in the intralesional nerve-sparing surgery group (mean 0.9). According to the literature, intralesional nerve-sparing surgery showed improvement of symptoms in 25–100%, maintenance in 13–38%, and deterioration in 8–38% of the patients [7, 16, 19, 21]. The proportion of patients who were asymptomatic at the final follow-up was 56–80% [25, 27, 28] (Table 5). In patients treated with intralesional nerve-sparing surgery following preoperative denosumab therapy, the proportion of patients who were asymptomatic at the final follow-up was 75–89% [27, 28] (Table 5). In the patients treated with the combination of denosumab therapy and embolization, the proportion of patients who were asymptomatic at the final follow-up was 83–100% [3, 15] (Table 6). In patients treated with embolization alone, the proportion of patients who were asymptomatic at the final follow-up was 67–100% [33, 35, 36] (Table 6). Thus, patients undergoing non-surgical treatment appear to have a better functional outcome than those who underwent intralesional nerve-sparing surgery, and our results confirm the data in the literature.

In this study, of the 11 women with sacral GCTB, 8 (73%) were under the age of 40 years, which is the childbearing age. There were no patients in either the intralesional nerve-sparing surgery and non-surgical treatment groups who were pregnant or delivered a baby. Because denosumab is teratogenic, female patients need to be contraceptive during denosumab administration (non-surgical treatment) [43, 44]. It is necessary to develop a drug that has fewer side effects than denosumab, can be used in pregnant women, and has the same effect as denosumab.

Our study has several limitations. First, this was a retrospective study with indication bias. Non-surgical treatment was performed more frequently in patients with large, centrally located tumors. Second, this study has the relatively short length of follow-up, especially for the non-surgical treatment group. Third, statistical analysis was not possible because of the small sample size. A well-designed randomized controlled trial with long-term follow-up is required to determine the optimal treatment for sacral GCTB. However, randomized controlled trials on sacral GCTB are quite difficult to conduct because sacral GCTB is very uncommon. To our knowledge, this is the first comparative study of patients with sacral GCTB who underwent intralesional nerve-sparing surgery or non-surgical treatment.

## Conclusions

The local recurrence rate was 44% in the intralesional nerve-sparing surgery group, and tumor control was achieved in all patients in the non-surgical treatment group. Non-surgical treatment has a similar risk of complications to intralesional nerve-sparing surgery and has better functional outcomes than intralesional nerve-sparing surgery. However, intralesional nerve-sparing surgery is the only option for achieving a disease-free condition for sacral GCTB. Non-surgical treatment seems to be a possible treatment option for GCTB of the sacrum. Based on our results, the decision on the choice of treatment for sacral GCTB could be discussed between the surgeon and patient based on the tumor size and location, considering that surgery can cure in over 50% of the patients, compared to the possibility of a non-surgical treatment that cannot achieve a disease-free status over time. In the future, it will be necessary to conduct a randomized clinical trial using a multicenter prospective collaborative study.

## Data Availability

The datasets generated, analyzed, or both during the present study are not publicly available because of privacy problems, but are available from the corresponding author upon reasonable request.

## Abbreviations

CT: computed tomography
GCTB: giant bone tumor of the bone
MRI: magnetic resonance imaging

## References

[1] Flanagan AM, Larousserie F, O’Donnell PG, Yoshida A. Giant Cell Tumour of Bone. In: The WHO Classification of Tumours Editorial Board. WHO classification of tumours, 5th ed: Soft Tissue and Bone Tumours. Lyon: IARC; 2020. p. 440–6.

[2] Campanacci M, Baldini N, Boriani S, Sudanese A (1987). Giant-cell tumor of bone. J Bone Joint Surg Am.

[3] Ji T, Yang Y, Wang Y, Sun K, Guo W (2017). Combining of serial embolization and denosumab for large sacropelvic giant cell tumor: case report of 3 cases. Medicine (Baltimore).

[4] Llauger J, Palmer J, Amores S, Bagué S, Camins A (2000). Primary tumors of the sacrum: diagnostic imaging. AJR Am J Roentgenol.

[5] Manaster BJ, Graham T (2003). Imaging of sacral tumors. Neurosurg Focus.

[6] Ruggieri P, Mavrogenis AF, Ussia G, Angelini A, Papagelopoulos PJ, Mercuri M (2010). Recurrence after and complications associated with adjuvant treatments for sacral giant cell tumor. Clin Orthop Relat Res.

[7] Thangaraj R, Grimer RJ, Carter SR, Stirling AJ, Spilsbury J, Spooner D (2010). Giant cell tumour of the sacrum: a suggested algorithm for treatment. Eur Spine J.

[8] Guo W, Ji T, Tang X, Yang Y. Outcome of conservative surgery for giant cell tumor of the sacrum. Spine (Phila Pa 1976). 2009;34:1025–31. 10.1097/BRS.0b013e31819d4127 19404178

[9] Lim CY, Liu X, He F, Liang H, Yang Y, Ji T, et al. Retrospective cohort study of 68 sacral giant cell tumours treated with nerve-sparing surgery and evaluation on therapeutic benefits of denosumab therapy. Bone Joint J. 2020;102-B:177–85. 10.1302/0301-620X.102B2.BJJ-2019-0813.R1 32009426

[10] Zhao Y, Tang X, Yan T, Ji T, Yang R, Guo W. Risk factors for the local recurrence of giant cell tumours of the sacrum treated with nerve-sparing surgery. Bone Joint J 2020;102-B:1392–8. 10.1302/0301-620X.102B10.BJJ-2020-0276.R1 32993346

[11] Chawla S, Henshaw R, Seeger L, Choy E, Blay J-Y, Ferrari S (2013). Safety and efficacy of denosumab for adults and skeletally mature adolescents with giant cell tumour of bone: interim analysis of an open-label, parallel-group, phase 2 study. Lancet Oncol..

[12] Palmerini E, Chawla NS, Ferrari S, Sudan M, Picci P, Marchesi E (2017). Denosumab in advanced/unresectable giant-cell tumour of bone (GCTB): for how long?. Eur J Cancer.

[13] Tsukamoto S, Mavrogenis AF, Kido A, Errani C (2021). Current concepts in the treatment of giant cell tumors of bone. Cancers (Basel).

[14] He S-H, Xu W, Sun Z-W, Liu W-B, Liu Y-J, Wei H-F (2017). Selective arterial embolization for the treatment of sacral and pelvic giant cell tumor: a systematic review. Orthop Surg.

[15] Puri A, Gupta SM, Gulia A, Shetty N, Laskar S. Giant cell tumors of the sacrum: is non-operative treatment effective? Eur Spine J 2020. 10.1007/s00586-020-06650-x 33106943

[16] Domovitov SV, Chandhanayingyong C, Boland PJ, McKeown DG, Healey JH (2016). Conservative surgery in the treatment of giant cell tumor of the sacrum: 35 years’ experience. J Neurosurg Spine.

[17] Moran D, Zadnik PL, Taylor T, Groves ML, Yurter A, Wolinsky J-P (2015). Maintenance of bowel, bladder, and motor functions after sacrectomy. Spine J.

[18] Langevelde KV, Vucht NV, Tsukamoto S, Mavrogenis AF, Errani C. Radiological assessment of Giant cell tumour of bone in the sacrum: from diagnosis to treatment response evaluation. Curr med Imaging. 2021. 10.2174/1573405617666210406121006 33845749

[19] Turcotte RE, Sim FH, Unni KK. Giant cell tumor of the sacrum. Clin Orthop Relat Res 1993;:215-21. 8504603

[20] Balke M, Henrichs MP, Gosheger G, Ahrens H, Streitbuerger A, Koehler M (2012). Giant cell tumors of the axial skeleton. Sarcoma..

[21] Chen K-H, Wu P-K, Chen C-F, Chen W-M (2015). Zoledronic acid-loaded bone cement as a local adjuvant therapy for giant cell tumor of the sacrum after intralesional curettage. Eur Spine J.

[22] Chen Z, Yang Y, Guo W, Yang R, Tang X, Yan T (2018). Therapeutic benefits of neoadjuvant and post-operative denosumab on sacral giant cell tumor: a retrospective cohort study of 30 cases. J BUON.

[23] Kollender Y, Meller I, Bickels J, Flusser G, Issakov J, Merimsky O (2003). Role of adjuvant cryosurgery in intralesional treatment of sacral tumors. Cancer..

[24] Li G, Fu D, Chen K, Ma X, Sun M, Sun W (2012). Surgical strategy for the management of sacral giant cell tumors: a 32-case series. Spine J.

[25] Martin C, McCarthy EF (2010). Giant cell tumor of the sacrum and spine: series of 23 cases and a review of the literature. Iowa Orthop J.

[26] van der Heijden L, van de Sande MAJ, van der Geest ICM, Schreuder HWB, van Royen BJ, Jutte PC (2014). Giant cell tumors of the sacrum--a nationwide study on midterm results in 26 patients after intralesional excision. Eur Spine J.

[27] Wang J, Du Z, Yang R, Tang X, Yan T, Guo W (2020). Analysis of clinical outcome for adolescent patients undergoing conservative nerve-sparing surgery based on the proposed resection classification for sacral giant cell tumor. J Clin Neurosci.

[28] Xu W, Wang Y, Wang J, Yang X, Liu W, Zhou W (2017). Long-term administration of bisphosphonate to reduce local recurrence of sacral giant cell tumor after nerve-sparing surgery. J Neurosurg Spine..

[29] Yang Y, Li Y, Liu W, Xu H, Niu X (2018). A nonrandomized controlled study of sacral giant cell tumors with preoperative treatment of denosumab. Medicine (Baltimore).

[30] Zhang R-Z, Ma T-X, Qi D-W, Zhao M, Hu T, Zhang G-C (2019). Short-term preoperative denosumab with surgery in unresectable or recurrent giant cell tumor of bone. Orthop Surg.

[31] Sung HW, Kuo DP, Shu WP, Chai YB, Liu CC, Li SM (1982). Giant-cell tumor of bone: analysis of two hundred and eight cases in Chinese patients. J Bone Joint Surg Am.

[32] Niu X, Yang Y, Wong KC, Huang Z, Ding Y, Zhang W (2019). Giant cell tumour of the bone treated with denosumab: how has the blood supply and oncological prognosis of the tumour changed?. J Orthop Translat.

[33] Chuang VP, Soo CS, Wallace S, Benjamin RS (1981). Arterial occlusion: management of giant cell tumor and aneurysmal bone cyst. AJR Am J Roentgenol.

[34] Hosalkar HS, Jones KJ, King JJ, Lackman RD (2007). Serial arterial embolization for large sacral giant-cell tumors: mid- to long-term results. Spine..

[35] Lackman RD, Khoury LD, Esmail A, Donthineni-Rao R (2002). The treatment of sacral giant-cell tumours by serial arterial embolisation. J Bone Joint Surg Br.

[36] Lin PP, Guzel VB, Moura MF, Wallace S, Benjamin RS, Weber KL (2002). Long-term follow-up of patients with giant cell tumor of the sacrum treated with selective arterial embolization. Cancer..

[37] Nakanishi K, Osuga K, Hori S, Hamada K, Hashimoto N, Araki N (2013). Transarterial embolization (TAE) of sacral giant cell tumor (GCT) using spherical parmanent embolic material superabsorbant polymer microsphere (SAP-MS). Springerplus..

[38] Balke M, Campanacci L, Gebert C, Picci P, Gibbons M, Taylor R (2010). Bisphosphonate treatment of aggressive primary, recurrent and metastatic Giant cell tumour of bone. BMC Cancer.

[39] Onishi H, Kaya M, Wada T, Nagoya S, Sasaki M, Yamashita T (2010). Giant cell tumor of the sacrum treated with selective arterial embolization. Int J Clin Oncol.

[40] Gottfried ON, Schmidt MH, Stevens EA (2003). Embolization of sacral tumors. Neurosurg Focus.

[41] Tang X, Guo W, Yang R, Tang S, Ji T (2009). Risk factors for blood loss during sacral tumor resection. Clin Orthop Relat Res.

[42] Chawla S, Blay J-Y, Rutkowski P, Le Cesne A, Reichardt P, Gelderblom H (2019). Denosumab in patients with giant-cell tumour of bone: a multicentre, open-label, phase 2 study. Lancet Oncol.

[43] Bussiere JL, Pyrah I, Boyce R, Branstetter D, Loomis M, Andrews-Cleavenger D (2013). Reproductive toxicity of denosumab in cynomolgus monkeys. Reprod Toxicol.

[44] Okamatsu N, Sakai N, Karakawa A, Kouyama N, Sato Y, Inagaki K (2017). Biological effects of anti-RANKL antibody administration in pregnant mice and their newborns. Biochem Biophys Res Commun.

